# Reporting Horizon Scanning Studies: Prototype Development Study

**DOI:** 10.2196/88253

**Published:** 2026-04-16

**Authors:** Sonia Garcia Gonzalez-Moral, Megan Fairweather, Sarah Khan, Ross Fairbairn, Gill Norman

**Affiliations:** 1 NIHR Innovation Observatory, Population Health Sciences Institute, Newcastle University, The Catalyst Room 3.12, 3 Science Square Newcastle Helix, Newcastle upon Tyne, England, NE4 5TG, United Kingdom, 0044 (0191) 2082262

**Keywords:** horizon scanning, reporting checklist, health care innovations, medical technologies, reporting guidelines

## Abstract

**Background:**

Horizon scanning identifies weak signals of innovation to anticipate future developments, providing strategic value for health care decision-making. Unlike evidence synthesis, it addresses emerging and uncertain areas but lacks standardized reporting guidance, limiting transparency, consistency, and impact. Inconsistent terminology and poorly described methods hinder comparability and uptake of findings.

**Objective:**

This study aimed to develop a prototype reporting checklist and glossary to support structured, transparent, and reproducible reporting of horizon scanning methods in health care innovation.

**Methods:**

A multidisciplinary working group of horizon scanning, evidence synthesis, and information specialists was convened at the National Institute for Health and Care Research Innovation Observatory, the UK national horizon scanning center, in May 2024. Using the PRISMA-ScR (Preferred Reporting Items for Systematic Reviews and Meta-Analyses extension for Scoping Reviews) checklist as a framework, 4 workshops were held to adapt relevant reporting items and build a prototype checklist. Internal validation was conducted on 17 eligible Innovation Observatory reports (2017‐2024), with scoring to assess item coverage. Items were then refined, classified as mandatory or optional, and finalized through consensus.

**Results:**

Of 46 outputs screened, 17 (37%) met the inclusion criteria. In total, 42.9% (15/35) of the checklist items achieved 50% or more coverage; 57.1% (20/35) scored less than 50%. The final 35-item checklist (n=31, 88.6% single items and n=4, 11.4% multipart items) includes 28 (80%) mandatory and 7 (20%) optional items. Four novel components were introduced: interest holder description; horizon, innovation, population, data source, and interest holder scope framework; technology characteristics; and integration of the political, economic, social, technological, legal, environmental, and demographic factors framework.

**Conclusions:**

This is the first checklist to standardize reporting in health technology horizon scanning. While this checklist prototype has been developed internally, external validation involving multidisciplinary experts via a Delphi process and a scoping review is planned. Its adoption can enhance transparency, reproducibility, and strategic impact, strengthening this methodological field.

## Introduction

### Background

Horizon scanning refers to the structured identification and analysis of weak signals, trends, drivers of change, wild cards, and discontinuities, enabling anticipation of future developments [1]. It belongs to the broader field of futures studies, which is concerned with exploring, modeling, and preparing for alternative future scenarios. Although horizon scanning has been widely adopted across diverse sectors, its application in the medical sciences is relatively recent [2]. The National Institute for Health and Care Research (NIHR) Innovation Observatory uses horizon scanning to systematically detect weak signals and trends of innovative health care technologies and support future decision-making processes about them [3].

Unlike evidence synthesis, understood as the process of systematically identifying, appraising, and integrating findings from multiple studies on a specific topic to generate comprehensive, reliable, and policy- or practice-relevant conclusions [4], horizon scanning focuses on the systematic process of detecting and collecting early signs and weak signals of potentially important developments that, in this context, may be interpreted as innovative health care technologies [5]. However, horizon scanning currently lacks standardized reporting guidance, particularly for key aspects of the horizon scanning process such as the identification, selection, and prioritization of emerging health technologies. Furthermore, a recent review of horizon scanning practices in medical technologies, diagnostics, and digital health recommended the use of dedicated reporting standards in light of the inconsistent use of terminology in the field [6]. This lack of clarity impedes the dissemination and utility of horizon scanning outputs—such as published reports and research articles—and contributes to research inefficiencies, including duplication and underuse of findings [7].

Horizon scanning is more likely to be used to widely explore the “unknowns,” being those more or less certain and definitively less defined, on emerging areas of innovation across diverse domains. Using the Rumsfeld matrix as a conceptual framework and using it to compare horizon scanning with other well-established methodologies such as evidence synthesis, we could argue that, while evidence synthesis is akin to examining the visible tip of an iceberg (“known knowns”), horizon scanning focuses on what lies beneath the surface (“unknown knowns” and “known unknowns”) [8].

While horizon scanning is recognized as a methodological approach in its own right, it requires clear principles and structured processes to ensure rigor and consistency. Despite its growing use in health and policy contexts, the field remains underserved by methodological frameworks, reporting standards, or dedicated guidance. This absence of consensus risks limiting the transparency, comparability, and overall impact of horizon scanning outputs. Reporting guidelines specify a minimum set of elements that should be included in research publications and have been shown to enhance methodological transparency and promote the use of research findings [9]. To our knowledge, no standard reporting guidelines currently exist to support the transparent and consistent documentation of horizon scanning activities in the health care domain. Given the increasing role of horizon scanning in health care decision-making—both in the United Kingdom and internationally—this study aimed to address this gap by developing the first reporting checklist for horizon scanning methods in medical innovation.

### Aim and Objectives 

This study aimed to initiate the standardization of reporting practices for horizon scanning methods and tools, thereby enhancing the transparency and reproducibility of such approaches.

Our objectives included the development of a conceptual framework for horizon scanning research question formulation and query translation and the development of a preliminary checklist specifying essential and optional elements that should be transparently reported in horizon scanning outputs and a glossary to support consistent interpretation and reduce ambiguity in terminology. Further work will support finalization of the checklist, incorporating the experience of those who used this preliminary version.

### Scope of This Checklist

This checklist is intended for application to horizon scanning outputs, including both articles published in academic journals and reports published on institutional websites. The checklist is developed to provide detailed and transparent information about the application of horizon scanning methods to medical health technologies. Intended users of this checklist include authors of such reports, peer reviewers of manuscripts submitted to journals for publication, and journal editors when assessing such manuscripts and methods.

## Methods

### Overview

A working group consisting of 4 horizon scanning specialists, 2 evidence synthesis specialists, and 2 information specialists was convened at the NIHR Innovation Observatory in May 2024. Participants were selected based on demonstrable expertise in horizon scanning, evidence synthesis methodology, and information science. Their disciplinary backgrounds spanned medical sciences, biosciences, and information science, and their professional experience included conducting systematic reviews, advanced information retrieval, and horizon scanning across medicines, medical devices, diagnostics, and digital health technologies. The group reflected methodological and professional diversity, encompassing varied levels of seniority and experience working on projects for multiple stakeholder groups. All members had established publication records in relevant methodological or applied domains, providing evidence of recognized subject matter expertise.

Four structured workshops, each lasting approximately 2 hours, were conducted between May 2024 and October 2024 to define objectives, iteratively refine methodological components, and develop the checklist. Prior to these sessions, an initial planning meeting was held in which the group established the study aims, scope, procedural approach, and decision-making processes that would guide the workshop series. During the workshops, proposed items and revisions were discussed collectively, and decisions were reached through structured deliberation, with agreement determined either through consensus where possible or, when consensus was not achievable, through majority agreement informed by methodological rationale and supporting evidence from the literature.

Given the similarity of broad scoping reviews with horizon scanning methods, we decided to use the PRISMA-ScR (Preferred Reporting Items for Systematic Reviews and Meta-Analyses extension for Scoping Reviews) as a framework to develop a checklist for reporting items relevant to horizon scanning reports [9]. During the first and second workshops, the group assessed the relevance of each PRISMA-ScR checklist item against the methodological and topic characteristics of horizon scanning reports and produced a modified checklist prototype for horizon scanning studies.

For the internal validation of the prototype, we applied the checklist to outputs produced by the Innovation Observatory between 2017 and 2024. Each team member in the working group was assigned a set of randomly selected reports to screen for suitability and, if applicable, score those reports that used horizon scanning methods for the identification of innovative health care technologies against our checklist prototype. Following the screening process, 17 reports were deemed suitable for scoring. The rest were excluded as they were not horizon scanning reports. Each checklist item was marked as “yes” (included), “no” (not included), or not applicable (“N/A”) if the item was not relevant to that specific paper. Following completion of the scoring exercise, one team member (MF) combined the scores for each item and calculated a final percentage score by dividing the score by the maximum possible score (“N/A” items were not included in the maximum score).

In the third and fourth workshops, the working group reviewed the scores, proposed additional customization of items to address issues relevant to horizon scanning based on the scoring exercise, and achieved consensus on whether the items should be made mandatory or optional. The resulting checklist prototype was uploaded to the Open Science Framework for dissemination.

### Ethical Considerations

This study did not involve human participants, patient data, or identifiable personal information. The work consisted solely of methodological development activities conducted by professionals in their occupational roles and analysis of publicly available institutional reports. According to institutional and national research governance guidance, this type of study does not constitute human subjects research and therefore did not require review or approval by an institutional review board or research ethics committee [10].

All procedures were conducted in accordance with applicable institutional, national, and international standards for research integrity and publication ethics [11].

## Results

### Overview

**Figure 1.**
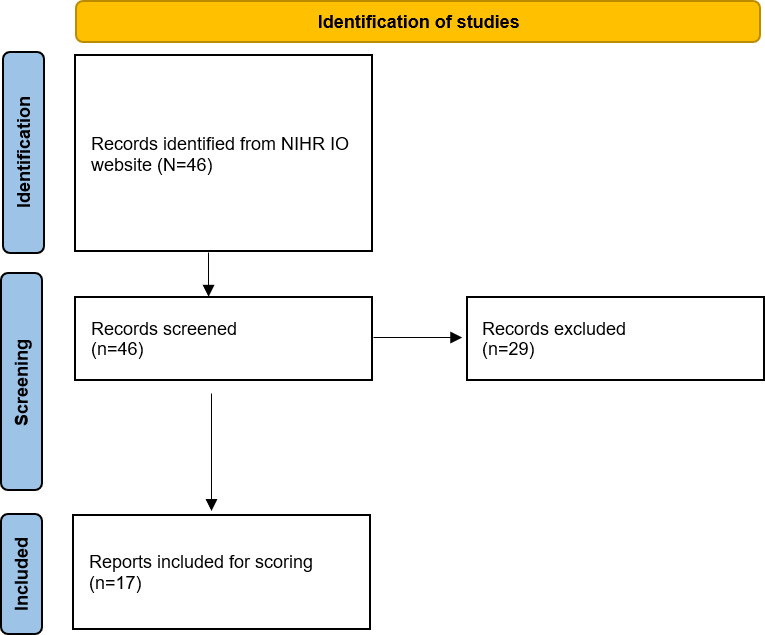
PRISMA (Preferred Reporting Items for Systematic Reviews and Meta-Analyses) flow diagram depicting included and excluded reports. IO: Innovation Observatory; NIHR: National Institute for Health and Care Research.

A total of 46 outputs published between 2017 and 2024 were downloaded from the NIHR Innovation Observatory website by a team member (Oleta Williams). In total, 37.0% (n=17) of the horizon scanning reports met the criteria for inclusion in this assessment and were assessed against the checklist. The remaining reports were not horizon scanning reports and were not suitable for this assessment. The reasons for exclusion were being only an abstract, a methods paper, or a presentation (not a report). Figure 1 presents the PRISMA flowchart for the identification of studies.

A total of 42.9% (15/35) of the items achieved a final score between 50% and 100%, whereas 57.1% (20/35) of the items scored under 50%. Table 1 presents a breakdown of these items by their total final score.

Next, based on the working group members’ experience, and in line with the objectives of this study, the items were rated through agreement as optional or mandatory. An optional item meant that the information required was not always possible to disclose due to a number of reasons that include technology stage of development, stakeholder agreements, availability of the information, or relevancy of the item in the context of the horizon scanning scope. These items start with an “If appropriate” statement to indicate their optionality. A mandatory item was deemed of the utmost importance for the conduct of horizon scanning methods and interpretation of results. These needed to be present at all times to achieve transparency in the retrieval and filtration of the signals and replicability of methods, avoid bias in the results, and achieve accountability and impact and overall increased rigor in the final horizon scanning output.

The resulting checklist contains 35 items (n=31, 88.6% single items and n=4, 11.4% that include multiple subitems), of which 28 (80%) were deemed mandatory and 7 (20%) were deemed optional (items 15, 16, 17b, 19, 20, 21, and 23). Some subsections, such as “Synthesis methods” (items 14a and 14b) in the “Methods” section and “Weak signal selection” (items 17a and 17b) in the “Results” section, present more than 1 item per section, with only item 17b considered optional. The sections “Discussion” (items 24a to 24d) and “Registration and protocol” (items 25a to 25c) also include multiple subitems and are all considered mandatory.

This checklist includes 4 new essential components not found in the PRISMA-ScR checklist. These are “Interest holder description” item 4 in the “Introduction” section; “Scope” item 6 in the “Methods” section; “Technology characteristics” item 19 in the “Results” section; and the introduction of the political, economic, social, technological, legal, environmental, and demographic factors (PESTLED) framework in item 24a in the “Discussion” section.

**Table 1.** Scoring checklist items by the number of times the item was present in the horizon scanning reports assessed.

Checklist item name (number)	Total final score (%)
Items that scored >50%	
Rationale (3)	100
Eligibility criteria (7)	100
Single source of information characteristics (18)	100
Discussion (24a)	100
Information sources (8)	94
Support (26)	94
Results by intended purpose or outcome (21)	90
Technology characteristics (19)	88
Discussion (24d)	88
Weak signal selection (17a)	81
Objectives (5)	76
Interest holder (4)	63
Data items (12)	60
Title (1)	53
Competing interests (27)	50
Items that scored <50%	
Discussion (24b)	46
Selection process (10)	46
Abstract (2)	41
Discussion (24c)	38
Search strategy (9)	38
Scope (6)	38
Availability of data, code, and other materials (28)	36
Data collection process (11)	33
Synthesis methods (14b)	33
Synthesis methods (14a)	30
Reporting bias assessment (15)	20
Weak signal selection (17b)	9
Effect measures (13)	0
Certainty assessment (16)	0
Risk of bias in studies (20)	0
Reporting missing data (22)	0
Certainty of evidence (23)	0
Registration and protocol (25a, 25b, and 25c)	0

### Interest Holder Description

In the context of health technology horizon scanning, an interest holder (often called a stakeholder) refers to any individual, group, or organization that has an interest in or may be affected by the development, adoption, use, or regulation of a health technology.

Key types of interest holders in this context may be the following:

- Patients and patient advocacy groups—interested in access to and the effectiveness and safety of new and innovative health technologies
- Clinicians and health care providers—interested in clinical utility, integration into practice, and outcomes
- Health technology developers and manufacturers—concerned with market potential, regulation, and innovation uptake
- Regulators and health authorities—interested in safety, efficacy, and compliance with regulatory standards or the early identification of gaps in current regulatory frameworks
- Policymakers and health system planners—focused on broader system impact and public health implications
- Researchers and academics—interested in evidence generation, gaps, and methodological development
- Health technology assessment (HTA) bodies—use horizon scanning to inform evaluation and prioritization

Interest holders contribute by identifying unmet needs and emerging technologies, understanding key eligibility criteria based on needs or gaps, informing prioritization criteria, providing early feedback on clinical relevance and implementation barriers, and supporting evidence appraisal and decision-making. Their involvement ensures that the scanning process is contextually relevant, anticipatory, and aligned with real-world health system priorities.

### Horizon Scan Scope: Horizon, Innovation, Population, Data Source, and Interest Holder Framework for Scoping Health Technology Horizon Scans

To support interpretability and decision relevance, horizon scanning reports should explicitly describe the contextual parameters that shape the scan, including its purpose, decision setting, intended users, policy or clinical domain, and analytic perspective. This is the purpose of applying the horizon, innovation, population, data source, and interest holder (HIP-D/I) scoping framework, which provides a structured approach to define the scope of horizon scanning projects targeting innovative health care technologies through the following 5 key dimensions. Clear specification of these elements enables readers to understand how signals were identified, interpreted, and prioritized and supports appropriate comparison across horizon scanning outputs:

- Horizon—horizon scanning studies should define their temporal scope (eg, short-, medium-, or long-term horizon) as part of the methodological description and within item 6 (“Scope”) of the checklist through the HIP-D/I framework as the selected time horizon influences the types of signals identified, their interpretation, and their relevance for decision-making contexts. Time horizon is categorized based on technologies’ developmental proximity to market launch: emerging (early-stage concepts), transitional (in precommercial or pilot phases), and imminent (near market entry and postmarket surveillance). This helps determine the level of readiness, the sources that should be searched based on the maturity of the evidence, and the time frame for potential impact.
- Innovation—focuses on identifying what is novel or disruptive about the technology compared to current standards of care. This includes technological, procedural, or conceptual innovations that may transform clinical practice or health system delivery.
- Population—specifies the target patient group or population segment that the technology is intended to benefit. Clarifying the population helps align horizon scanning priorities with unmet clinical needs and equity considerations.
- Data sources—emphasizes the importance of identifying weak signals of innovation. Given the developmental stage of many technologies, relevant data may be found in nontraditional sources such as preprints, patents, clinical trial registries, venture capital investments, and early-stage conference presentations. Data sources and horizon are intrinsically related.
- Interest holder—as defined above, they provide a contextual element to the results of the horizon scanning, enabling greater and immediate impact.

Together, these 5 dimensions support a systematic scoping process that enables transparent and reproducible methods for the identification and prioritization of transformative health technologies.

### Technology Characteristics

In horizon scanning studies of health care innovation, providing a detailed description of the identified, filtered, and prioritized technologies is essential to ensure that outputs are meaningful and actionable for decision-makers. Unlike systematic reviews—where the unit of analysis is typically the study and its outcomes—horizon scanning primarily focuses on the identification of innovative technologies and often uses the technology and its characteristics (eg, trial phase or technology readiness levels) as the primary unit of interest. Detailed technology descriptions capture critical attributes such as intended use, mechanism of action, stage of development, anticipated benefits, implementation challenges, and potential impact on health systems.

This level of detail serves several purposes: it supports informed prioritization, facilitates future assessments (eg, HTA or regulatory review), enhances transparency and reproducibility, and enables strategic planning for health system readiness.

To align with these goals, the reporting checklist was adapted to shift the focus from evidence appraisal, as seen in the PRISMA-ScR checklist, to the detection of weak signals, in keeping with the purpose and definition of horizon scanning [12]. Table 2 presents the prototype checklist, including item descriptions and corresponding document sections.

**Table 2.** Final checklist items

Section and subsection	Item number	Item description
Title		
Not applicable	1	Identify the report as a horizon scan or other alternative terms used to refer to horizon scanning outputs, for example, landscape or pipeline analysis or report or broader umbrella terms such as foresight analysis
Abstract		
Not applicable	2	See the adaptation of the PRISMA^a^ 2020 for Abstracts checklist for horizon scanning reports
Rationale	3	Describe the rationale for the horizon scan in the context of existing knowledge, unmet needs, policy, or emerging innovation
Introduction		
Interest holders	4	Disclosure of interest holder names and brief function or role in decision-making
Objectives	5	Provide an explicit statement of the objectives or questions that the horizon scan addresses
Methods		
Scope	6	Consider using the HIP-D/I^b^ framework
Eligibility criteria	7	Specify the inclusion and exclusion criteria for the horizon scan
Information sources	8	Specify all databases, registers, websites, organizations, reference lists, and PPIE^c^ and expert solicitation and consultation, as well as any other sources searched or consulted to identify signals; specify the date when each source was last searched or consulted
Search strategy	9	Present the full search strategies for all databases, registers, and websites, including any filters and limits used; report if any AI^d^ tool was used for creating or running the searches and provide the name and version number of the tool
Selection process	10	Specify the methods used to decide whether a signal met the inclusion criteria of the horizon scan, including how many reviewers screened each record and each report retrieved; whether they worked independently; and, if applicable, details of automation tools used in the process
Data collection process	11	Specify the methods used to collect data from reports, including how many reviewers collected data from each report; whether they worked independently; any processes for obtaining or confirming data from third parties; and, if applicable, details of automation tools used in the process
Data items	12	List and define all data points sought; describe any assumptions made about any missing or unclear information
Effect measures	13	If appropriate to the technology readiness level or technology type (medicine, diagnostic tests, medical devices, or digital interventions), specify for each outcome the effect or performance measures (eg, risk ratio, mean difference, sensitivity or specificity, and ROC^e^ curve) used in the presentation of results
Synthesis methods	14a	Describe the processes used to decide which signals were eligible for each analysis (checklist item 7)
Synthesis methods	14b	Describe any methods required to prepare the data for presentation or analyses, such as handling of missing data or data conversions
Reporting bias assessment	15	If appropriate to the technology readiness level or technology type (medicine, diagnostic tests, medical devices, or digital interventions), state if a risk-of-bias assessment of the studies used for identification of signals will be performed and which tools will be used
Certainty assessment	16	If appropriate to the technology readiness level or technology type (medicine, diagnostic tests, medical devices, or digital interventions), describe any methods used to assess certainty (or confidence) in the body of evidence for an outcome using the GRADE^f^ approach
Results		
Weak signal selection	17a	Describe the results of the search and selection process, from the number of records identified in the search to the number of technologies included in the horizon scan, ideally using a flow diagram
Weak signal selection	17b	If appropriate, include a list in the appendix or a separate tab of technologies that might appear to meet the inclusion criteria but that were excluded and explain why they were excluded; these may be referred to as “other interesting technologies” that do not meet the criteria pre-established by interest holders but present some innovative aspects of interest
Single source of information characteristics	18	If appropriate, cite each included single source of information and present its characteristics
Technology characteristics	19	If appropriate for the scan scope, list the technology characteristics
Risk of bias in studies	20	If appropriate to the technology readiness level or technology type (medicine, diagnostic tests, medical devices, or digital interventions), present assessments of risk of bias for each included study
Results by intended purpose or outcome	21	If appropriate, present analyses by outcome, signal, and/or technology
Reporting missing data	22	Present missing data and how they have been dealt with in the analysis
Certainty of evidence	23	If appropriate to the technology readiness level or technology type (medicine, diagnostic tests, medical devices, or digital interventions), present assessments of certainty (or confidence) in the body of evidence for each outcome assessed using the GRADE approach
Discussion		
Not applicable	24a	Provide a general interpretation of the results in the context of other PESTLED^g^ factors
Not applicable	24b	Discuss any limitations of the data included in the horizon scan
Not applicable	24c	Discuss any limitations of the horizon scanning processes used (including language barriers—eg, only English language considered)
Not applicable	24d	Discuss the implications of the results for practice, policy, and future research
Other information		
Registration and protocol	25a	Provide registration information for the review, including register name and registration number, or state that the review was not registered
Registration and protocol	25b	Indicate where the review protocol can be accessed or state that a protocol was not prepared
Registration and protocol	25c	Describe and explain any amendments to the information provided at registration or in the protocol
Support	26	Describe sources of financial or nonfinancial support for the review and the role of the funders or sponsors in the review
Competing interests	27	Declare any competing interests of review authors
Availability of data, code, and other materials	28	Subject to agreement with the stakeholders, report which of the following are publicly available and where they can be found: template data collection forms, data extracted from the included studies, data used for all analyses, analytic code, and any other materials used in the review

a PRISMA: Preferred Reporting Items for Systematic Reviews and Meta-Analyses.

b HIP-D/I: horizon, innovation, population, data source, and interest holder.

c PPIE: public and patient involvement and engagement.

d AI: artificial intelligence.

e ROC: receiver operating characteristic.

f GRADE: Grading of Recommendations Assessment, Development, and Evaluation.

g PESTLED: political, economic, social, technological, legal, environmental, and demographic factors.

### PESTLED Framework

Incorporating the PESTLED framework into the discussion section of a horizon scan on emerging health care technologies enriches the report by situating innovations within a structured macroenvironmental context, thereby highlighting how regulatory dynamics, economic constraints, social and demographic values, and environmental considerations intersect with technological development and diffusion. For instance, a strategic environmental scan of Iranian public hospitals demonstrated that PESTLED-guided analysis enabled the identification of external pressures, including economic sanctions, shifts in disease patterns, and governance issues, which informed adaptive strategies for equitable resource allocation and service delivery [13]. Similarly, scoping reviews applying PESTLED-type analysis to electronic health record implementation revealed how political, economic, sociocultural, and legal dimensions shape adoption trajectories and reveal implementation challenges in low- and middle-income settings [14]. By framing horizon scan findings within the 7 domains of this framework, authors can present a reproducible and comparative analytic lens that directly links emerging technologies to broader system drivers; aids in anticipating barriers to and enablers of adoption; and enhances the strategic relevance of the report for policymakers, funders, and health system planners.

The full proposed checklist is shown in Table 2 and Checklist 1.

The checklist is available for download [15]. A full item-by-item description with text excerpts as examples is provided in Checklist 2. Checklist 1 provides the checklist form for completion at the reporting writing stage. A glossary of terms for ease of interpretation of the checklist items is provided in Checklist 3.

## Discussion

### Principal Findings

This study introduced what is, to the best of our knowledge, the first horizon scanning checklist designed to enhance transparency, reproducibility, and methodological rigor in the reporting of health technology horizon scanning studies. It presents the results of preliminary work involving analysis of our own reports and in-depth discussions among expert practitioners within the UK horizon scanning center. The checklist outlines identified key features that should be systematically reported to enable comprehensive interrogation and interpretation of intelligence derived from horizon scanning. When our reports were assessed against the checklist, just over one-third of the items (15/35, 42.9%) achieved a final score above 50%, whereas the remaining 57.1% (20/35) of the items were insufficiently reported. This variation in reporting highlights a lack of standardization in current horizon scanning outputs and demonstrates the need for a structured reporting framework. Six checklist items (13, 16, 20, 22, 23, and 25) received a score of 0, meaning that they were not reported in any of the included reports assessed. Some of these items might not be relevant to all technology readiness levels and should not always be reported. For example, a horizon scan in the early horizon that uses patents and preclinical studies to identify trends and signals of innovative technologies will not present sufficient data on outcomes or the strength of the evidence to warrant risk-of-bias or certainty assessments. Furthermore, although it is strongly recommended that a horizon scan follow a robust, pre-agreed protocol, the highly strategic nature of some horizon scans makes a publicly available protocol unfeasible due to confidentiality agreements with stakeholders. Notably, many of these features are already represented in reports from the UK national horizon scanning center, highlighting both their relevance and feasibility. This finding demonstrates the need for more consistent and comprehensive reporting across all horizon scanning studies to support full transparency, comparability, and utility of the findings.

Key strengths observed across well-reported items included the consistent articulation of rationale, eligibility criteria, and technology characteristics, crucial elements for supporting decision-making. However, core methodological domains such as search strategy, selection process, synthesis methods, and discussion of limitations were frequently underreported or absent. These gaps limit the transparency and replicability of horizon scanning studies, potentially reducing their utility for decision-makers involved in regulatory, reimbursement, or research prioritization pathways.

To address these issues, the study team developed a prototype 35-item checklist (including multicomponent subitems), of which 28 (80%) were designated as mandatory and 7 (20%) were designated as optional. These designations were based on the expert consensus of internal team members and reflected both practical constraints and methodological requirements. It is worth stressing that the checklist introduces 4 essential components not found in existing reporting frameworks such as the PRISMA-ScR: interest holder description, scope (HIP-D/I framework), technology characteristics, and PESTLED framework. These additions are fundamental to horizon scanning, which (unlike systematic reviews) uses the technology as the unit of analysis rather than the study or its outcomes. A detailed description of the identified, filtered, and prioritized technologies is critical to enable strategic planning, future assessments, and timely decision-making.

Furthermore, this checklist reflects the exploratory and anticipatory nature of horizon scanning. The emphasis is on the detection of weak signals, in alignment with the goals of identifying transformative technologies ahead of market entry. This reframing supports the unique objectives of horizon scanning, particularly in cases in which conventional data may be unavailable and insights must be drawn from diverse and sometimes non–peer-reviewed sources such as clinical trial registries, patent databases, preprints, and expert consultation.

Horizon scanning is increasingly used by a range of stakeholders (regulators, HTA bodies, policymakers, clinicians, and technology developers) as a forward-looking, market intelligence, and decision support tool [16-19]. Given the variability in how horizon scanning is defined and implemented across organizations, the proposed checklist may provide a common foundation for improving the transparency, comparability, and rigor of horizon scanning practices in health care innovation.

Our study has several limitations. First, the development of the checklist prototype relied exclusively on internal experts from the NIHR Innovation Observatory. While the team possesses extensive experience in conducting horizon scans across a variety of health technologies, stakeholders, and time horizons, we recognize that this internal scope may limit the generalizability of the findings. To address this, we plan to undertake a formal external validation using a scoping review and a multistage adapted Delphi process to achieve broader consensus on item relevance, structure, and applicability across diverse horizon scanning contexts. Second, the checklist scoring was based on a relatively small sample of 17 reports published since 2017. Given the niche nature of horizon scanning in health care, this limited sample size may have influenced the ranking of optional vs mandatory items. Future application of the checklist to a larger set of outputs will help mitigate this constraint and may refine item prioritization. Finally, wild cards did not appear in the analyzed reports because the sample consisted of applied horizon scanning outputs focused on near- to midterm health technologies, where evidence sources and stakeholder priorities typically emphasize plausible and actionable innovations rather than low-probability, high-impact events. Therefore, their absence reflects the empirical characteristics and scope of the sampled material, not a conceptual exclusion from the checklist framework, which is designed to accommodate any signal type if reported but more specifically weak signals and trends.

### Conclusions and Next Steps

This study presents the development of a prototype checklist designed to improve the transparency, consistency, and methodological clarity of horizon scanning reports for health care innovation. The checklist was created in response to a recognized gap in reporting standards [6], drawing on elements from the PRISMA-ScR framework [9] to accommodate the broad and exploratory nature of horizon scanning studies, similar to those of broad scoping reviews [20].

The checklist provides a structured foundation for reporting horizon scanning outputs, facilitating more robust and reproducible methods, and supporting stakeholder engagement in early decision-making processes. Its adoption has the potential to contribute to the standardization of horizon scanning practices across organizations, enhance the strategic value of these outputs in innovation management, and provide horizon scanning methods of greater scientific rigor.

Next steps include conducting an external validation using a multistage Delphi process following a rapid scoping review involving a diverse group of horizon scanning experts and end users. This will help refine and potentially increase the checklist items, confirm their relevance across different settings, and promote broader adoption. If widely implemented, this checklist can serve as a key tool for strengthening the scientific basis of horizon scanning and advancing its role in health policy and innovation planning.

## Supplementary material

10.2196/88253 Checklist 1 Checklist of reporting items for horizon scanning studies (version 1.4).

10.2196/88253 Checklist 2 Checklist of reporting items for horizon scanning studies: Examples.

10.2196/88253 Checklist 3 Checklist items glossary of terms.

## Abbreviations

HIP-D/I: horizon, innovation, population, data source, and interest holder
HTA: health technology assessment
NIHR: National Institute for Health and Care Research
PESTLED: political, economic, social, technological, legal, environmental, and demographic factors
PRISMA-ScR: Preferred Reporting Items for Systematic Reviews and Meta-Analyses extension for Scoping Reviews
