# Supplementary material for: Reporting Horizon Scanning Studies: Prototype Development Study
Source: J Med Internet Res. 2026 Apr 16;28:e88253. doi: 10.2196/88253 (PMC13086060; doi:10.2196/88253)
Supplement: Checklist 3 [file jmir-v28-e88253-s003.docx]

| **Concept** | **Definition** |
| --- | --- |
| Analysis | Exploration of overall results by pooling information from extracted data to identify trends. Could include, for example, formal statistical analysis or narrative synthesis. |
| Automation tools | Automation tools are used to speed up the process of horizon scanning searching, screening or data extracting. There are no currently available tools that replace human intervention in any of the mentioned horizon scanning tasks. Automation tools may be used to assist with some manual processes. |
| Certainty | The degree of confidence that an estimate of the effect is correct, indicating the level of certainty that such estimate can reliably inform a particular recommendation or decision. |
| Competing interests | A competing interest, also known as a conflict of interest (COI), is when a secondary interest may influence the professional judgment of a primary interest. |
| Data collection forms | It usually a detailed spreadsheet where columns describe the data point and rows the technology characteristics that fir within each data point. Collection is usually undertaken manually. |
| Data point | A discrete unit of information about a particular characteristic or feature extracted from sources and collected in a dataset (usually a spreadsheet). A data point may contain numerical or alphabetic values, and these may be in the form of single words or entire sentences. |
| GRADE | Grading of Recommendations Assessment, Development and Evaluation (GRADE) is a widely adopted tool for assessing the certainty of evidence. This tool is used to produce a ‘summary of findings’ table which provides an assessment of certainty for each outcome. |
| Interest holder | Anyone who has an interest in a project, outcome, or decision. This can include stakeholders, who also have a direct influence or involvement in the decision-making process. |
| HIP-D/I  framework | A framework used to structure a research question which can be used to guide a horizon scan scope. The following concepts are recommended for consideration: Horizon, Innovation, Population, Data source  **Horizon** refers to how far into the future the horizon scan is looking at, it often involves defining the technology readiness level or the time limits in which the searches are framed. This usually determines the sources that are searched in line with the horizon.  **Innovation** refers to the technology characteristics or technology features that are known and of interest. These may be very varied and include aspects such as portability, remote connectivity, artificial intelligence components, rapidity, but also, stage of development or technology readiness levels, etc…  **Population** broadly describes the most likely population that would benefit from that technological innovation and may be defined by health state or condition.  **Data source** defines the most likely sources to find signals of innovation. These will depend on other concepts such as the horizon.  **Interest holder** they provide a contextual element to the results of the horizon scanning, enabling greater and immediate impact. |
| PPIE | Patient and Public Involvement and Engagement (PPIE) is a valuable process which is beneficial for patient and public trust of research. Public involvement in research means research that is done ‘with’ or ‘by’ the public, not 'to', 'for' or 'about' them so that patients or others with relevant experience contribute to how research is carried out. Engagement involves the sharing of knowledge and findings from research to the patients and public as well as an opportunity for discussion so feedback can be retrieved to optimise future work. |
| Review protocol | A detailed plan that outlines the methods and objectives for a research project such as horizon scan |
| Risk of bias | The likelihood that elements of a study's design or execution compromises the validity of study findings, as well as the conclusions drawn from the research. By systematically examining the design, conduct, and reporting of studies, risk of bias tools support researchers to determine the trustworthiness of the evidence. |
| Scope | The scope of a horizon scanning project involves the definition of the objectives of the project, the research questions, the inclusion and exclusion criteria and stage of development or time horizon as well as the sources that will be searched. Furthermore, scoping for a horizon scanning project should involve multiple interest holders and horizon scanning analysts and information specialists. It usually is undertaken iteratively with a final scope approval and sign off. The scope of a horizon scanning project will determine what will be included and analysed which makes this step highly important for the relevancy of the final results. The scope may also include deliverables (report, dashboard, presentations, publications, etc); timelines and resource capacity. |
| Screened | The process in which information sources/records are assessed against the remit or inclusion/exclusion criteria |
| Signal | In horizon scanning for health technologies, a signal may be interpreted as an emerging trend or indication of a new healthcare technology, this may include existing healthcare technologies used in different populations or for different healthcare outcomes. |
| Single source of information | An individual source from where weak signals have been identified and data has been collected. These may be a published or unpublished study, a news item, funding call, patent documents or social media sources. |
| Source | An origin of information or data. Horizon scanning sources include news, conferences, clinical trial registries, funding databases, patent databases, grey literature sources, policy, regulatory news and regulations and bibliographic databases amongst others |
| PESTLE | A foresight framework used for analysing external factors that can influence the technology landscape: Political, Economic, Social, Technological, Legal, and Environmental |
| Technology Readiness Level (TRLs) | A common framework for defining the stage of development of a technology. Ranges from TRL1 (earliest stage) to TRL9 (latest stage). |
| Weak signal | Weak signals are early indications of development of an event with low probability and therefore described as ‘weak’ |
